# Eleonora’s falcon trophic interactions with insects within its breeding range: A systematic review

**DOI:** 10.1515/biol-2025-1187

**Published:** 2025-11-05

**Authors:** Ioanna Angelidou, Thomas Hadjikyriakou, Alexander N. G. Kirschel, Angeliki F. Martinou, Helen Elizabeth Roy, Anastasios Saratsis, Georgios Karris

**Affiliations:** Department of Environment, School of Environment, Ionian University, Zakynthos, GR-29100, Greece; Laboratory of Vector Ecology and Applied Entomology, Joint Services Health Unit, British Forces Cyprus, RAF Akrotiri, Limassol, BFPO57, Cyprus; Enalia Physis Environmental Research Centre, Nicosia, CY-2101, Cyprus; Akrotiri Environmental Education Centre, Akrotiri, 4640, Cyprus; Department of Biological Sciences, University of Cyprus, Nicosia, CY-1678, Cyprus; Climate and Atmosphere Research Center, The Cyprus Institute, Nicosia, 2121, Cyprus; UK Centre for Ecology & Hydrology, Wallingford, OX10 8BB, England, UK; Centre for Ecology and Conservation, University of Exeter, Penryn, Cornwall, TR10 9FE, England, UK; Veterinary Research Institute, Hellenic Agricultural Organisation Dimitra, Thermi, 570 01, Greece

**Keywords:** Eleonora’s falcon, *Falco eleonorae*, forage, insects, pre-breeding and breeding period

## Abstract

Eleonora’s falcon (*Falco eleonorae*) has a unique ecology that makes it ideal for studying predator–prey dynamics. Its diet shifts seasonally, with insects dominating the non-breeding and pre-laying periods, and birds becoming the main prey during chick-rearing. This systematic review explores the falcon’s foraging behavior during the pre-breeding and breeding phases within its global breeding range, emphasizing insect prey. Following PRISMA guidelines, the review includes studies from ten Mediterranean countries and the Canary Islands. Literature was sourced from Web of Science, Google Scholar, PubMed, and Scopus, covering both peer-reviewed and grey literature up to 2024. From 18 scientific publications and personal observations, 120 insect species and morphospecies from 47 families were recorded as prey. Coleoptera, Hymenoptera, and Hemiptera were the most frequent insect orders. Migratory species such as *Acherontia atropos*, *Anax parthenope*, and *Anax ephippiger* were also documented. This review contributes valuable knowledge for future studies on the dietary ecology of Eleonora’s falcon and similar raptors.

## 1 Introduction

Eleonora’s falcon (*Falco eleonorae* Géné, 1839) is included in Annex I of the European Directive 2009/147/EC. It is protected by local, regional, national, and European frameworks [1], and it constitutes a priority species for conservation [2,3,4,5]. It is a highly specialized bird of prey [6], with unique phenology in its trans-equatorial year-round movements, its foraging behavior, and the timing of reproduction [7,8]. It breeds in colonies on the sea cliffs of islands and on rocky islets of the Mediterranean Sea and North-western African coast, and coastal waters of the Atlantic [9,10,11]. During winter, it primarily migrates to Madagascar, with some individuals reaching East and Southeast Africa, covering up to 18,000 km annually between breeding and wintering grounds [1,3,10,12,13]. As the latest raptor breeder in the Northern Hemisphere [8,14,15], Eleonora’s falcon presents a unique adaptation. More specifically, it starts nesting in late summer (August–September), a period coinciding with the autumn migration of passerines, which constitute the primary prey for both adults and nestlings during the rearing period [2,4,8,16,17,18,19,20]. This adaptation is shared only with two closely related falcon species: the Sooty falcon (*Falco concolor*) [21,22,23] and the Eurasian hobby (*Falco subbuteo*) [22]. However, Eleonora’s falcon is unique in its primary diet, as it mainly preys on insectivorous bird species [24,25]. Unlike the Sooty falcon, which has a more varied diet including small birds and insects, and the Eurasian hobby, which also hunts small birds and large insects, Eleonora’s falcon is highly specialized as an aerial predator of large, winged insects (>10 mm in length) throughout the year [26]. This insectivorous diet is maintained during all stages of its life cycle, including wintering grounds, migration, and breeding sites, up until the egg-laying period [15,20,27]. Notably, as the breeding season progresses, Eleonora’s falcon reduces its insect consumption and shifts increasingly toward hunting insectivorous birds, which form the majority of its diet during this period [28].

The prevalence of insects in the diet of Eleonora’s falcon is evident in the pre-breeding season, throughout its distribution across southern Europe [29]. During this time, birds can travel distances of up to 600 km from their breeding colonies in search of prey [29,30]. They visit a variety of habitats, including forests, phrygana (garigue), and arable lands, in search of insect prey [31]. In contrast, during the autumn period, their foraging behavior shifts. Falcons tend to stay closer to their breeding colonies, typically clustering around island and cliff sites [31]. At this time, they hunt in groups, primarily during the morning and evening hours [32], with an average foraging range of just 17 km from their nesting sites [29].

The diet of Eleonora’s falcon varies considerably in prey composition across its global range [13]. Many studies on feeding strategies in Eleonora’s falcon have investigated its hunting activity on migratory birds during the breeding season [13,28,33,34], neglecting the crucial contribution of insects, especially in certain habitats or during specific periods like the pre-breeding season. On the contrary, few studies have focused on insect hunting during the pre-breeding and breeding periods [15]. In the context of global environmental changes potentially driving rapid declines in insect abundance [35], this review presents the diet of Eleonora’s falcon during the pre-breeding and breeding periods within its global breeding range. Prey analysis might aid in detecting mismatches between the Eleonora’s falcon breeding activity and prey availability [15]. This study has three main objectives. First, it aims to compile and summarize current knowledge on the interactions between Eleonora’s falcon and insect prey within its global breeding range, using both systematic studies and scattered data from the literature. Second, it seeks to create a detailed list of insect species consumed by the falcon, which will serve as a reference point for future comparisons of diet composition across breeding sites worldwide. Third, the study addresses a key gap in the literature: despite extensive research on the falcon’s bird predation during the breeding season, little attention has been given to its foraging behavior during the pre-breeding and breeding periods, particularly with respect to insect consumption at nesting sites.

This review of the literature on Eleonora’s falcon–insect interactions is crucial, as insects can represent a significant part of the falcon’s diet. Understanding these interactions offers valuable insights into broader ecological dynamics, including how the species responds to changes in prey availability and environmental conditions. In light of ongoing habitat loss and environmental change, such a review can highlight potential risks to falcon populations, particularly those dependent on insects during the breeding season [36,37]. Ultimately, this work contributes to a deeper understanding of Eleonora’s falcon’s trophic relationships and supports the development of informed conservation strategies to protect both the species and its habitats.

## 2 Methods

### 2.1 Literature search

This systematic review followed the Preferred Reporting Items for Systematic Reviews and Meta-Analyses (PRISMA) 2020 guidelines (Supplementary file 1) [38] and was conducted on February 6, 2024, using data available up to that year. Geographically, it covered the global breeding range of the target species, namely the Mediterranean Sea and the Canary Islands, including the countries of Algeria, Croatia, Cyprus, Greece, Italy, Morocco, Portugal, Spain, Tunisia, and Turkey. Records of foraging activity of Eleonora’s falcon during the pre-breeding and breeding period were incorporated, using a combination of search words to find both published articles and grey literature material (MSc/PhD theses, reports, etc.) in four international bibliographic databases (Web of Science, Google Scholar, PubMed, and Scopus). We placed no restrictions on the year of the publication. The following search words in quotation marks were used: (“Eleonora’s falcon” OR “*Falco eleonorae*”) AND (insect OR insectivorous OR invertebrate OR forag* OR “nocturnal hunting” OR hunting OR prey OR pre-breeding OR “pre breeding” OR breeding) AND (Mediterranean OR Greece OR Cyprus OR Spain OR “Atlantic coast” OR Morocco OR “Canary Islands” OR “Northern Africa” OR Aegean OR Italy OR Canaries OR Algeria OR “Balearic Islands” OR Croatia OR Tunisia OR Sicily OR Sardinia OR “Aegean islands” OR Crete OR Turkey OR Azores) (Supplementary file 2).

Our objective was to identify countries with available data on Eleonora’s falcon’s foraging behavior during the pre-breeding and breeding period, highlight regions lacking such data, determine key insect taxa contributing to its diet across different areas, and evaluate gaps in understanding the species’ dietary ecology during this period.

### 2.2 Data extraction and selection criteria

**Figure 1:**
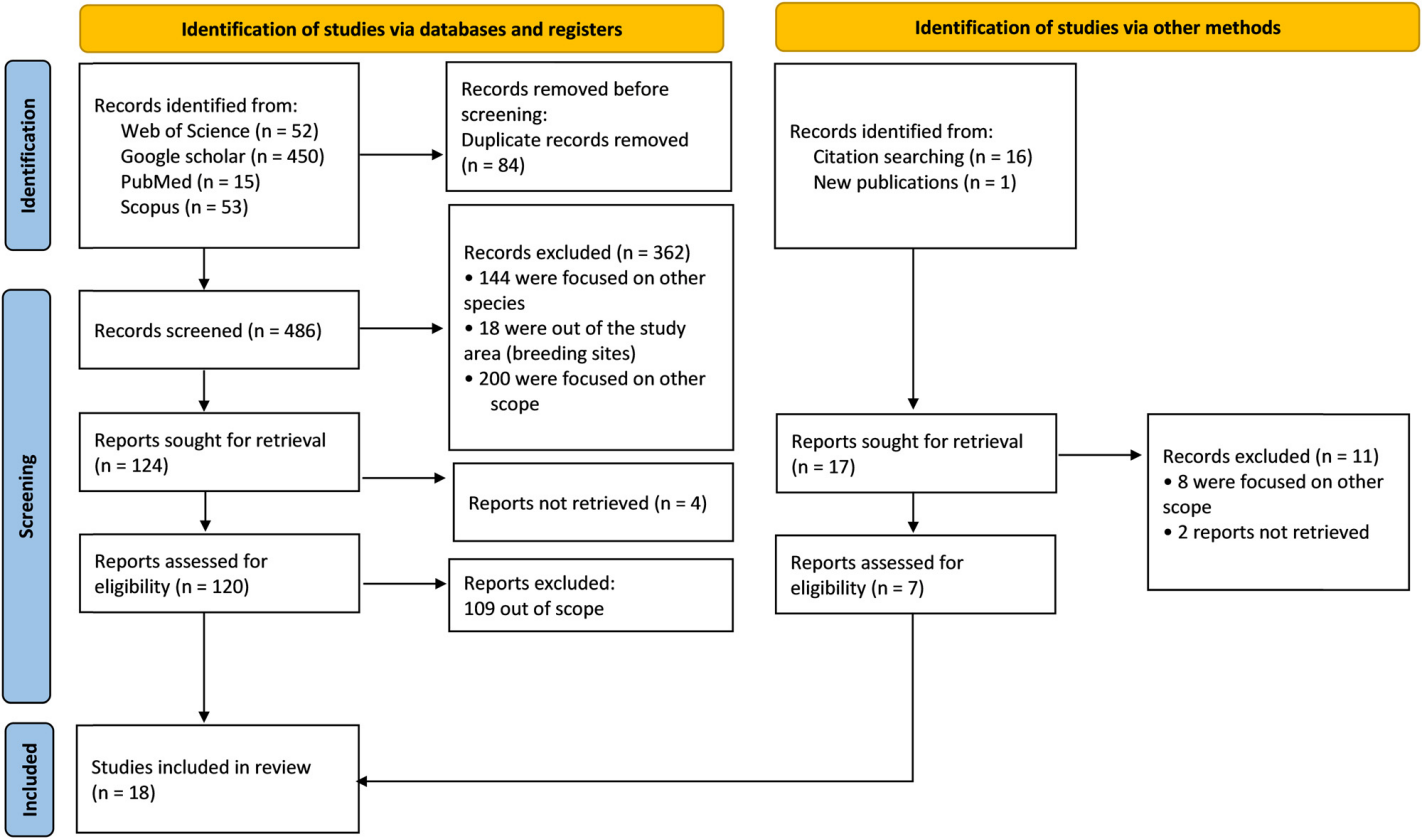
Simplified illustration of the record selection flow diagram following the PRISMA systematic search.

Retrieved records were first screened to exclude duplicates. Subsequently, titles and abstracts of all unique records were screened for their relevance to the scope of the review by the first and last author. This was achieved based on the following list of exclusion criteria (Figure 1; Supplementary file 2): (i) studies concerning a different species than Eleonora’s falcon; (ii) studies reporting data beyond the study area, such as wintering areas; (iii) studies reporting results outside of the scope of the review question (i.e. not on foraging activity of Eleonora’s falcon and invertebrate hunting); and (iv) languages other than those spoken in countries where Eleonora’s falcon breeds. When a document’s relevance could not be determined from the abstract and/or title, the full text was screened. Full texts, including relevant citations therein, were then retrieved where possible and evaluated according to the same criteria listed above by the first and last author. All data extracted were listed in Table 2 and Supplementary file 3.

### 2.3 Study area map and graphical visualisation

**Figure 2:**
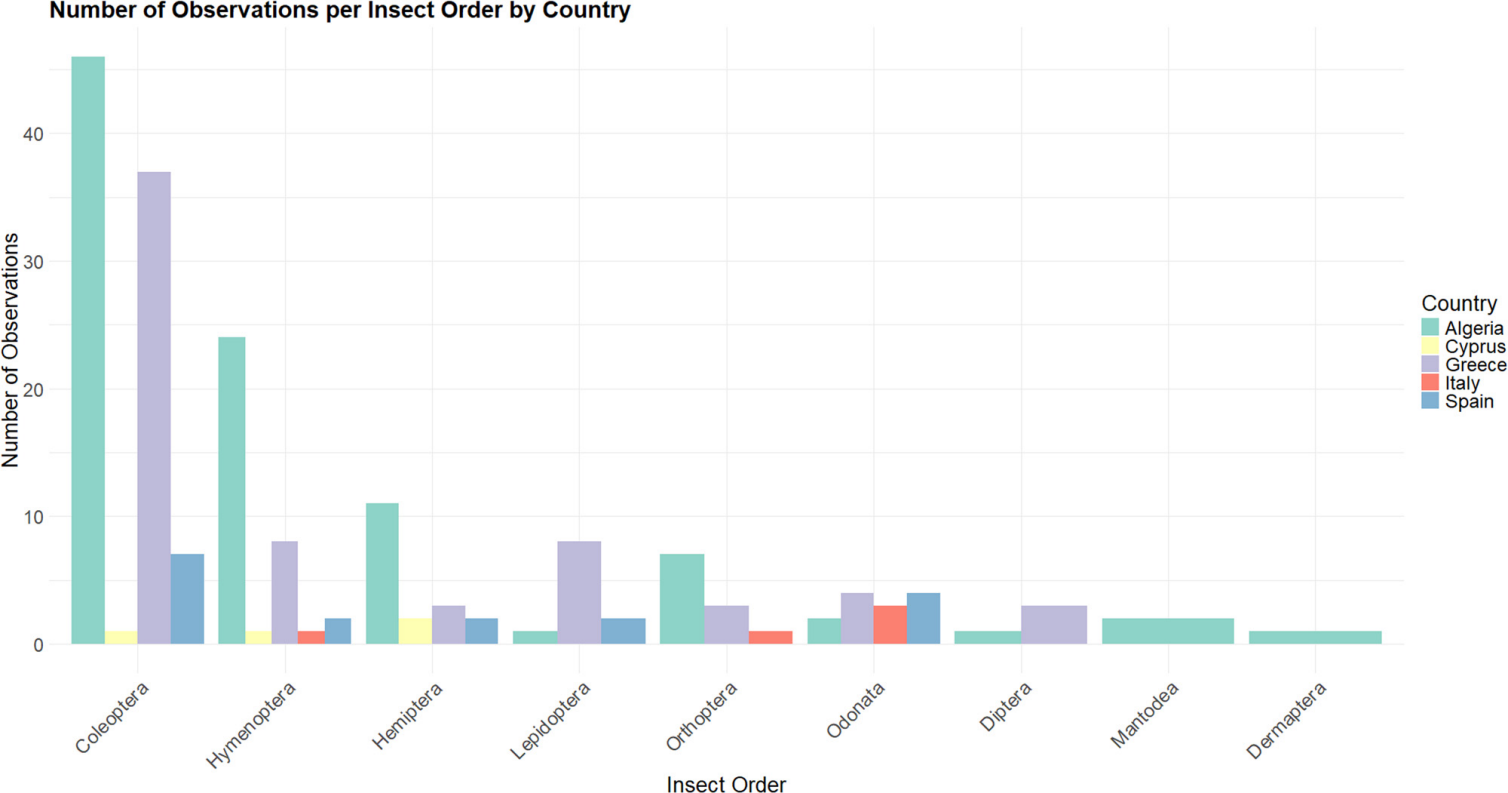
Number of observations of insect orders as Eleonora’s falcon prey by country.

Spatial data delineating the breeding sites of the species were obtained upon request from BirdLife International and the *Handbook of the Birds of the World* [39] (Figure 2). The data that were provided in polygon format were imported into QGIS version 3.30.1 [40] for mapping. According to this dataset, the total breeding range covers approximately 121952.482 km².

Details about the insectivorous diet of Eleonora’s falcon during the pre-breeding (late May to early July) and breeding (mid–July to October) seasons in Algeria and Greece were gathered from existing literature sources [15,28]. Additionally, QGIS was used to map the study area, encompassing all countries where the species is known to breed. Shapefiles of administrative boundaries for Algeria, Cyprus, Croatia, Greece, Italy, Morocco, Portugal, Spain, Turkey, and Tunisia were downloaded from the OpenDataSoft platform [41] (Figure 2).

All plots were generated in RStudio (version 1.1.4) using the ggplot2 package [42].

## 3 Results

### 3.1 General results

A literature search across four databases, Web of Science (*n* = 52), Google Scholar (*n* = 450), PubMed (*n* = 15), and Scopus (*n* = 53), returned a total of 570 results for countries in the Mediterranean Basin and the Atlantic Ocean. Among these, 84 were duplicates and subsequently removed (Figure 1). After screening titles and abstracts based on our selection criteria, the number of relevant records was reduced to 124. However, for four of these records, we were unable to retrieve the full text. Data on the dietary ecology of Eleonora’s falcon, specifically its consumption of insects during the pre-breeding and breeding periods, remain limited and fragmentary. Only 18 studies on this topic were identified, published between January 1938 and 2 September 2024. Country-specific examples include works by

- Italy: Massa [43], Corso et al. [44] (cited in Corso [45])
- Greece: Ristow [46], Xirouchakis et al. [15]
- Spain: De León et al. [47], Owens and Riddiford [48], Urios et al. [49], Mayol et al. [50]
- Algeria: Bakour and Moulaï [28], Samraoui et al. [13].

Two scientific articles, by Vaughan [51] and Mellone et al. [4], refer to earlier historical works by Munn (1925; 1931–32), Moltoni (1937), v. Wettstein [52], Stresemann [53], Uttendorfer (1948), Palau-Camps (1956–57), Cano (2001), and Belenguer et al. (2004). Of these, only v. Wettstein [52], who noted Eleonora’s falcon feeding on dung beetles, and Stresemann [53], who described birds preying on wasps (both in Aegean Greece), were available online. Additionally, Corso [45] and Corso et al. [44] cited the work of Walter [2], Spina (1992), Lo Cascio (1999), and Ristow [46], all highlighting dragonfly predation by Eleonora’s falcon in Italy. However, the original works by Spina (1992) and Lo Cascio (1999) could not be retrieved.

Ristow and Wink [33] identified aerial invertebrates as the falcon’s primary prey, including a wide range of insect orders such as Coleoptera, Hymenoptera, Diptera, Orthoptera, Hemiptera, Odonata, and Lepidoptera. Urios et al. [49] highlighted the consumption of *Polyphylla fullo* and cited Cano (2001) and Belenguer et al. (2004), who reported *Melolontha melolontha* as part of the diet. Mayol et al. [50], building on the work of Adrover and Mas [54] and others, documented additional taxa such as *Cerambyx cerdo*, *Pentodon algirus*, *Cicada orni*, *Acherontia atropos*, and species from the genus *Messor*. These records, including seven identified in September 2024 through supplementary sources [2,33,45,46,52,53,54], significantly enhance the existing knowledge on the diversity and ecological relevance of invertebrate prey consumed by *Eleonora’s falcon* (Table 1).

**Table 1:** Invertebrate prey of Eleonora’s falcon identified in selected studies

Insect order	Example species/genera	Source(s)
Coleoptera	*Buprestidae*, *Polyphylla fullo*, *Melolontha melolontha*, *Cerambyx cerdo*, *Pentodon algirus*	Ristow and Wink [33], Urios et al. [49], Mayol et al. [50], Adrover and Mas [54]
Hymenoptera	*Camponotus*, *Pheidole*, *Tetramorium*, *Messor*	Ristow and Wink [33], Mayol et al. [50], Adrover and Mas [54]
Diptera	Not specified	Ristow and Wink [33]
Orthoptera	Not specified	Ristow and Wink [33]
Hemiptera	*Cicada orni*	Ristow and Wink [33], Mayol et al. [50], Adrover and Mas [54]
Odonata	Anisoptera, Zygoptera	Ristow and Wink [33], Mayol et al. [50], Adrover and Mas [54]
Lepidoptera	*Acherontia atropos*	Ristow and Wink [33], Mayol et al. [50], Adrover and Mas [54]

We review these findings here per country. In Italy, Massa [43] studied Eleonora’s falcon foraging behavior through direct observations and pellet collection on Lampedusa, the Pelagian Islands, and the Sicilian Channel and Aeolian Islands during October 1975 and 1976, while Corso et al. [44] (cited in Corso [45]) made direct observations from Lampedusa to Lampione in July 2009. In Greece, V.Wettstein [52] and Stresemann [53] analyzed stomach contents from samples collected in May 1935 and October 1942, respectively. Ristow [46] conducted a pellet study and a general prey study at nests in a large Eleonora’s Falcon colony on an islet off Crete in 1997 and from August to October 1988. In the same study, Ristow [46] analyzed stomach contents from eight deceased falcons collected between 1999 and 2001, as an additional supplement to the primary study. Pellets were also analyzed for intact insects by Xirouchakis et al. [15], in their study across 16 Eleonora’s falcon colony islets located in the northern, central, and southern Aegean from late May to mid-October in 2004, 2005, and 2006. Ristow and Wink [33] studied the diet of Eleonora’s Falcon from 1965 to 2001, focusing on passerine birds, on a rocky islet north of Crete, using direct observations and nest item examination. Insects were also collected but were not included in their analysis and results. In Algeria, Bakour and Moulaï [28] collected pellets from adult Eleonora’s falcons at three nesting colonies (Plane Island, Ronde Island, and Rachgoun Island) during the 2016 breeding season. Similarly, Samraoui et al. [13] gathered dietary samples from Kef Amor between 2010 and 2012. In Spain, De León et al. [47] examined Eleonora’s Falcon diet with a focus on bird prey through nest examinations during the 2000 and 2001 breeding seasons in the Canary Islands. However, a few insect species were also collected in their study and are included in this review. Owens and Riddiford [48], in their study on the bees and wasps of the Balearic Islands, noted the large numbers of Eleonora’s Falcons foraging on insects in the area from late May to early June (year not referred), as observed by birdwatchers (Table 2).

**Table 2:** Insect-based diet of Eleonora’s falcon at the breeding grounds, based on the literature search

Country	References and time framework of the studies	Direct observations	Pellet analysis	Nest-item examination	Stomach-content examination
Italy	Massa [43]: June 1975 and first half of May 1976; Corso [45] and Corso et al. [44]: July 2009	Odonata (3 species)	Hymenoptera (1 morphospecies)		Orthoptera (1 species)
		Orthoptera (1 species)			
Greece	Ristow [46]: 1997, August–October 1998 and on stomach samples collected in 1999–2001; Xirouchakis et al. [15]: Late May to mid-October 2004, 2005, 2006	Coleoptera (6 morphospecies)	Coleoptera (9 species and 18 morphospecies)	Coleoptera (3 morphospecies)	Coleoptera (6 morphospecies)
		Hemiptera (1 morphospecies)	Diptera (3 morphospecies)	Hemiptera (1 morphospecies)	Hemiptera (2 morphospecies)
		Hymenoptera (2 species and 2 morphospecies)	Hemiptera (2 morphospecies)	Hymenoptera (1 morphospecies)	Hymenoptera (2 species and 2 morphospecies)
		Lepidoptera (3 morphospecies)	Hymenoptera (3 morphospecies)	Lepidoptera (2 morphospecies)	Odonata (3 morphospecies)
		Odonata (2 morphospecies)	Lepidoptera (6 morphospecies)	Odonata (2 species)	Orthoptera (1 morphospecies)
		Orthoptera (1 morphospecies)	Odonata (3 morphospecies)	Orthoptera (1 species)	
			Orthoptera (3 morphospecies)		
Spain	De León et al. [47]: Breeding seasons of 2000, 2001; Owens and Riddiford [48] – this is a study on the Bees and Wasps of the Balearic Islands that referred to birdwatchers’ observations	Coleoptera (2 species)			
		Odonata (1 morphospecies)			
		Lepidoptera (1 species)			
Algeria	Bakour and Moulaï [28]: August, September and October 2016; Samraoui et al. [13]: September–October 2010, September–October 2011 and July–October 2012		Coleoptera (45 species and morphospecies)	Coleoptera (1 species)	
			Dermaptera (1 species)	Lepidoptera (1 species)	
			Diptera (1 morphospecies)	Odonata (1 species)	
			Hemiptera (1 species and 10 morphospecies)		
			Hymenoptera (13 species and 11 morphospecies)		
			Mantodea (2 morphospecies)		
			Odonata (1 species)		
			Orthoptera (5 species and 2 morphospecies)		
Cyprus	Vaughan [51] Referred to Walker (pers. corn.); personal observations [55] during pre-breeding period of 2023, 2024	Hymenoptera (1 morphospecies)			
		Hemiptera (Cicadas, 2 species)			
		Coleoptera (1 species)			

### 3.2 Trophic interactions of Eleonora’s falcon with insects

A total of nine insect orders were recorded: Coleoptera with 16 families and more than 59 species and morphospecies; Dermaptera with one species; Diptera with two families and at least two species and morphospecies; Hemiptera with seven families and at least 13 species and morphospecies; Hymenoptera with five families and more than 28 species and morphospecies; Lepidoptera with five families and at least five species and morphospecies; one family and two species of Mantodea; Odonata with three families and at least four species and morphospecies; and Orthoptera with four families and at least ten species and morphospecies (Sub-table). The most frequently recorded insect orders were Coleoptera, Hymenoptera, and Hemiptera; Mantodea and Dermaptera were recorded only in Algeria (Figure 2). There is one record from Cyprus indicating that Eleonora’s falcon feeds on Hymenoptera (such as flying ants). However, personal observations [55] on the Akrotiri Peninsula in Cyprus reveal that the species also feeds on *Lyristes gemellus* and *Cicada orni* (family Cicadidae) and *Anoxia* sp. (family Scarabaeidae).

**Figure 3:**
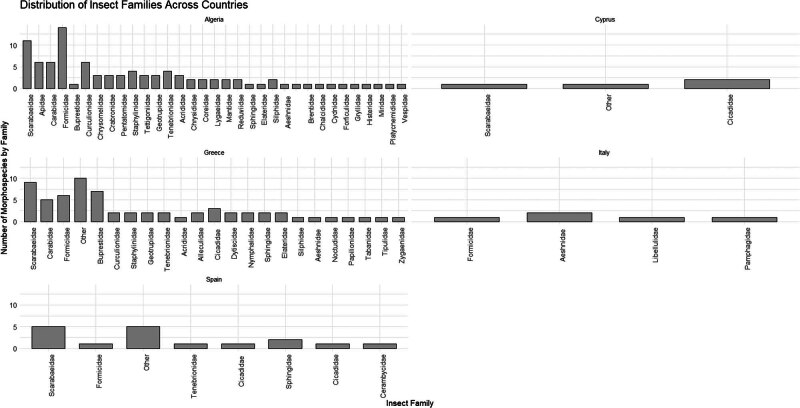
Insect families recorded from Algeria, Cyprus, Greece, Italy, and Spain. Columns “Other” represent general records, including categories like wasps, ants, or entries recorded only at the Order level.

The most frequently recorded insect families were Scarabaeidae (with 26 records; 13.9%), Formicidae (with 22 records; 11.76%), Carabidae (with 11 records; 5.88%), Curculionidae, and Buprestidae (with eight records each; 4.28% each). At a national level, the most frequently recorded insect families in Algeria were Formicidae (with 14 records), Scarabaeidae (with 11 records), Apidae, Carabidae, and Curculionidae (with six records each). In Greece, the most frequently recorded insect families were Scarabaeidae (with nine records), Buprestidae (with seven records), Formicidae (with six records), and Carabidae (with five records). Only a few families were recorded in Italy, i.e. Aeshnidae with two records, and Formicidae, Libellulidae, and Pamphagidae with one record each. In Spain, the most frequently recorded insect families were Scarabaeidae (with five records), Cicadidae, Sphingidae (with two records each), Cerambycidae, Formicidae, and Tenebrionidae were recorded once (Figure 3). Finally, in Cyprus, Cicadidae with two records, Scarabaeidae and Hymenoptera (family is not recorded) with one record.

**Figure 4:**
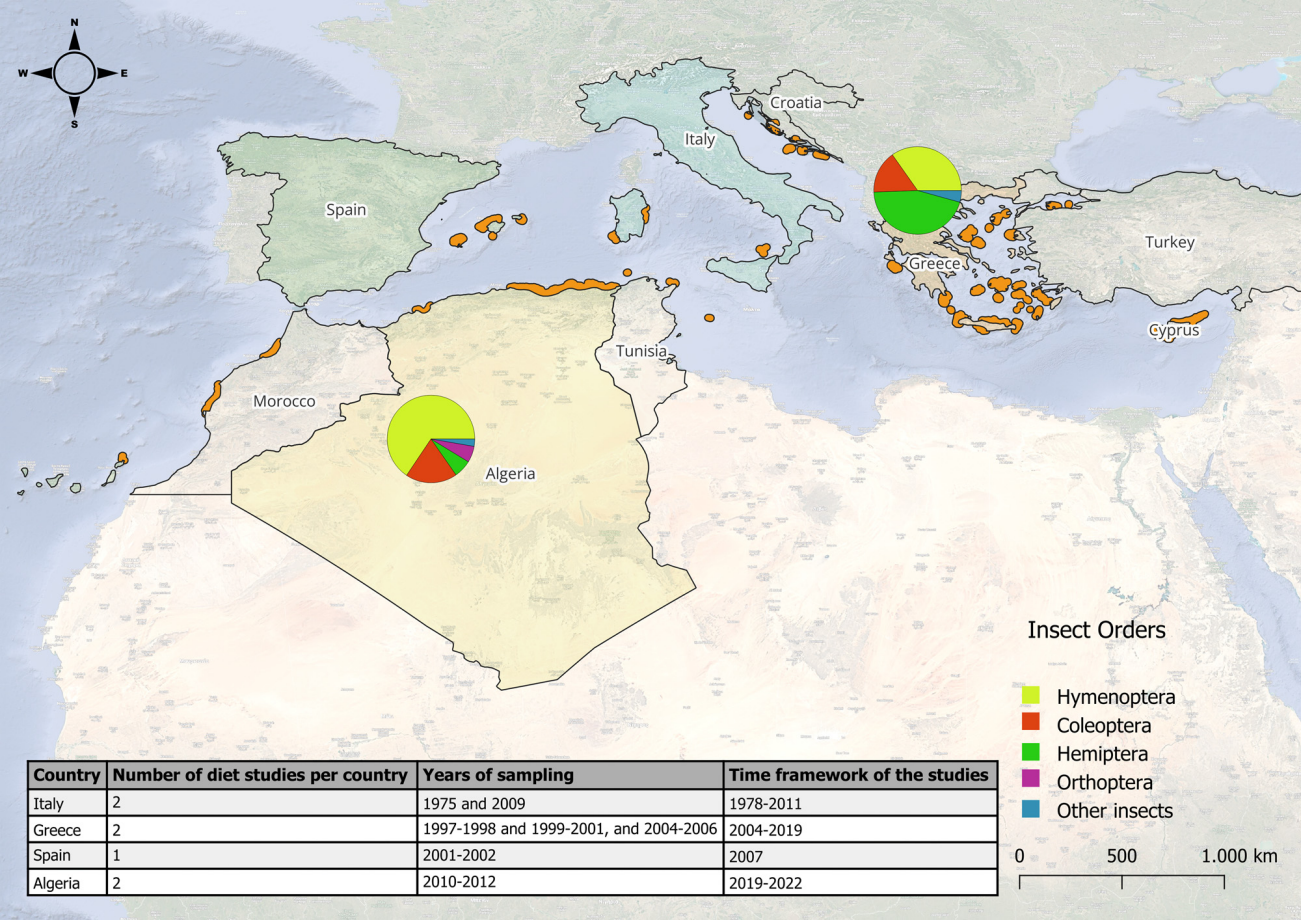
Map of Eleonora’s falcon feeding records during breeding season by district retrieved from previous studies; the breeding sites of the species are shown in orange are based on BirdLife International and Handbook of the Birds of the World [39]. Pies show the frequency of occurrence of insect prey in Greece (34.8% Hymenoptera, 15.8% Coleoptera, 45.1% Hemiptera, and 4.3% Other insects) and in Algeria (65.6% Hymenoptera, 19.1% Coleoptera, 6.6% Hemiptera, 6% Orthoptera, and 2.7% Other insects) based on the studies of Xirouchakis et al. [15] and Bakour and Moulaï [28], respectively. The table shows the number of diet studies per country, the year of sampling, and the time framework of the studies.

Regarding the frequency of the occurrence of insect prey at a national level, the study of Bakour and Moulaï [28] in Algeria/Western Mediterranean showed that *Gryllus* sp. occurred most frequently with 16.4%, followed by *Camponotus gestroi* with a frequency of 12.9%, *Tetramorium biskrensis* with 7.9% and *Pheidole pallidula* with 7.2%. The remainder of the insect prey taxa that made up the diet of Eleonora’s falcon occurred at lower frequencies, from 0.3 to 5.7%. In addition, the same study found that Hymenoptera were the most frequently represented insect order, followed by Coleoptera [28] (Figure 4). In contrast, Xirouchakis et al. [15] highlighted that Hemiptera (Cicadidae), with 45.1%, was the most frequent diet group in Greece/Eastern Mediterranean, followed by Hymenoptera, and specifically the Formicidae family, with 34.8% (Figure 4).

Furthermore, Ristow [46] found that Eleonora’s falcon in Crete/Greece feeds on Coleoptera, such as the *Carabus* sp. and *Calosoma sycophanta* (Carabidae), *Agabus* sp. (Dytiscidae), *Elater ferrugineus* (Elateridae), *Chalcophora* spp. (Buprestidae), as well as on some species of the subfamily Cetoniinae (e.g. *Potosia* sp.), Melolonthinae (Scarabaeidae), and Geotrupinae (Geotrupidae), and the families Silphidae, Staphylinidae, Alleculidae, Tenebrionidae, Buprestidae, and Curculionidae. Hymenoptera such as *Formica* sp., *Camponotus* sp., and *Messor* sp. (Formicidae), Hemiptera (Cicadidae), Lepidoptera, and more specifically the families Zygaenidae, Papilionidae, Nymphalidae, and Sphingidae, Odonata, and Orthoptera (Saltatoria) were also part of the species’ diet. In accordance with the previous findings, Xirouchakis et al. [15] reported that the species, across Aegean colonies, hunts on Hemiptera (Cicadidae), Hymenoptera (Formicidae), Coleoptera (Scarabaeidae, Carabidae, Buprestidae, Alleculidae, etc.), Odonata (Aeshnidae), Orthoptera (Acrididae), Diptera (Tipulidae, Tabanidae, etc.), and Lepidoptera (Nymphalidae, Noctuidae, Sphingidae).

In Italy and the Central Mediterranean, Massa [43] observed that the diet of the species comprised Orthoptera *Pamphagus ortolani* and Hymenoptera (Formicidae), while Corso [45] and Corso et al. [44] found it mainly consisted of the Odonata *Anax parthenope, Anax ephippiger* (Aeshnidae), and *Sympetrum fonscolombii* (Libellulidae). In Spain, and the Western Mediterranean, De León et al. [47] observed that the diet consisted primarily of *Acherontia atropos* (Sphingidae, Lepidoptera) and *Hegeter* sp. (Tenebrionidae, Coleoptera), while Mellone et al. [4] and Owens and Riddiford [48] reported that Eleonora’s falcon hunts Coleoptera *Melolontha melolontha* and *Polyphylla fullo*, and Anisoptera (Odonata). In addition, Urios et al. [49] reported that Eleonora’s falcon preys on Coleoptera, specifically *Polyphylla fullo* and *Melolontha melolontha,* while Mayol et al. [50] and Adrover and Mas [54] documented a broader insect diet. This includes representatives from Coleoptera (e.g*., Cerambyx cerdo, Polyphylla fullo, Pentodon algirus*), Hemiptera (e.g., *Cicada orni*), Lepidoptera (e.g., *Acherontia atropos*), Hymenoptera (notably the genus *Messor*), and Odonata (both *Anisoptera and Zygoptera*).

In Algeria, Bakour and Moulaï [28] reported that during the breeding season, Eleonora’s falcon feeds on a variety of invertebrates. These include Odonata (*Platycnemis* sp.) and Orthoptera species such as *Gryllus* sp*., Aiolopus strepens, Calliptamus barbarus, Decticus albifrons, Ochrilidia filicornis* and three species belonging to the Tettigoniidae family. Additionally, Dermaptera (*Forficula auricularia*), Mantodea (*Mantidae* sp. and *Mantis religiosa*), Hemiptera (Coreidae with two species, *Sehirus* sp., Lygaeidae with two species, *Miridae* sp., Pentatomidae with three species, Reduviidae with two species), and Diptera from the Suborder Brachycera were documented. The diet also comprised Coleoptera (*Anthaxia* sp., Carabidae with three species, *Licinus* sp. with two species, *Poecilus* sp., Chrysomelidae with three species, *Apion* sp., *Baris* sp., Curculionidae with three species, *Lixus* sp*., Otiorhynchus* sp., Elateridae with one species, *Hister* sp*., Amphimallon* sp*., Rhizotrogus* sp*., Aphodius* sp*., Euoniticellus* sp*., Geotrupes* sp. with three species, *Onthophagus* sp*., Polyphylla fullo, Potosia opaca, Protaetia* sp*., Phyllognathus silenus, Silpha granulata, Silpha* sp*., Atheta* sp*., Ocypus olens, Stenosis* sp., and Tenebrionidae with three species, but also insects from the Subfamilies Harpalinae, Pterostichinae with three species, Cetoniinae, Staphylininae, and Xantholinae). Regarding the Hymenoptera included in the species’ diet, they consisted of three species from the Apidae family and three from the Apoidea group, as well as one species from the Chalcididae family and two from Chrysididae. Other identified species were *Aphaenogaster testaceopilosa*, *Aphaenogaster sardoa*, *Aphaenogaster* sp., *C. gestroi*, *Camponotus* sp., *Camponotus truncatus*, and *Crematogaster scutellaris*. Additionally, one more species from the Formicidae family was documented, along with *Messor barbarus, Messor* sp*., Monomorium salomonis, Monomorium* sp*., Pheidole pallidula, Tetramorium biskrensis*, and one species from the Vespidae family. Moreover, Samraoui et al. [13] found in Algeria that the species also consumes *Acherontia atropos* (Lepidoptera), *Anax* sp. (Odonata), and *Oryctes nasicornis* (Coleoptera). In Cyprus, Vaughan [51] found that Eleonora’s falcon feeds on flying ants. Further observations in Cyprus have found the species feeding on cicadas (i.e. *Lyristes gemellus* and *Cicada orni*) and *Anoxia* sp. (family Scarabaeidae) (personal observations [55]; Sub-table). Vaughan [51], also drawing from literature, reported that Eleonora’s falcon preys on various invertebrates, including Odonata (Anisoptera), Orthoptera (e.g., *Dociostaurus maroccanus* and *Poecilimon cretensis*), Coleoptera (e.g., Carabidae, Buprestidae, Curculionidae, Tenebrionidae, and Scarabaeidae), Hymenoptera (wasps and ants), Hemiptera (cicadas), and Lepidoptera.

In summary, the most frequently recorded insect genera were *Camponotus* and *Messor*, both recorded in Algeria and Greece. *Acherontia atropos* and *Polyphylla fullo* were the individual species documented twice, in Algeria and Spain. All other insect species were recorded only once. Moreover, some of the identified prey insects are well-known for their migratory behavior, such as moths from the genus *Acherontia* (e.g., *Acherontia atropos*), which are resident in North Africa and regular summer migrants to Europe, as well as dragonflies from the genus *Anax* (e.g., *Anax parthenope* and *Anax ephippiger*) (Sub-table).

## 4 Discussion

Most raptor species across the globe leave their breeding grounds, often traveling in large flocks at predictable times and along consistent routes, to reach distant regions, sometimes on entirely different continents within the Nearctic and Palearctic realms. This includes two distantly related groups of diurnal raptors in the Orders Falconiformes and Accipitriformes, which are convergent in their morphology and general ecology [56]. Both Falconiformes and Accipitriformes include species that often coexist across various environments, while morphologically and ecologically similar species from these orders are likely to compete for shared resources in overlapping habitats [56,57]. As a result, sympatric species with similar ecological demands must find ways to reduce competition. One way this is accomplished is through dietary niche partitioning; understanding this overlap can discern how species allocate resources [58]. According to Gryz eand Krauze-Gryz [59] predators (including raptors) may present alternative feeding strategies, i.e. being either food specialists or opportunists, while at the same time, their diets change to reflect the prey availability and to avoid competition for food resources. Raptors preying on the same food category can avoid competition by hunting in different habitats [59]. For example, Eleonora’s falcon and Sooty falcon are two closely related species which primarily hunt aerial prey (birds and insects). Both species display delayed breeding phenology until late summer [6,22,29]. They feed their nestlings with migrating passerines during the peak of autumn migration, usually nest in dense aggregations on islands, and spend their non-breeding season primarily in Madagascar [6,21,22,23,60]. However, their distributions during the winter are largely non-overlapping, with Eleonora’s falcon found more commonly in humid regions [17,61,62], and Sooty falcon in drier habitat [22]. In the wintering grounds, Eleonora’s falcon is commonly found foraging in forests, feeding on termites, ants, and beetles, and near watercourses that attract insects such as dragonflies [61]. In addition, it is quite active at night, most often during moon illumination, while the use of artificial light sources aids hunting [57]. By contrast, Sooty Falcon is recorded hunting at night around flashlights in the capital of Madagascar where true bugs (Heteroptera–Hemiptera), beetles, and moths (Noctuidae, Pyralidae, and Sphingidae) were identified as prey items [57]. Similarly, various diurnal raptors have been observed to also forage at night, including other members of the genus *Falco*, increasing exploitative competition by sharing prey [57]. In addition to Eleonora’s falcon, which takes advantage of nocturnally migrating birds, Peregrine falcon appears to be successful during nocturnal hunting focused on disoriented birds [63].

Knowledge of the diet of raptors is important for understanding their biology, while diet studies are important both for management and conservation, with some management strategies now focusing on prey as a key element for maintaining populations of predator species of conservation concern [58,64]. Dietary assessment methods for raptors often include direct observation [58,65], camera placement at nest sites [12,58], examination of stomach contents [58], digestive tract flushing, forced vomiting, examination of fatty acid and isotope signatures, fecal analysis [51,65], and morphological investigation of pellets [58,65,66]. Nevertheless, most of the raptor diet studies focus on the breeding season as bird activity is concentrated around and at the nest, where prey remains are relatively easy to collect [64].

Focusing on Eleonora’s falcon, its survivorship is intimately linked to that of its prey [13]. However, most research on the species’ feeding strategies emphasizes its foraging and hunting behaviors during the breeding season, and particularly its reliance on migratory birds, such as passerines [28]. In contrast, relatively few studies have explored Eleonora’s falcon’s insect hunting during the pre-breeding and breeding periods [15]. As a result, knowledge of its insect-based diet at the breeding grounds remains limited (Table 2 and Sub-table).

Furthermore, the analyzed studies on insect–prey availability across countries did not all cover the same time period. Most studies did not fully capture the entire breeding cycle of Eleonora’s falcon; studies that did not specify the study period were not referenced here. Specifically, in Italy, studies covered early summer (June 1975) and mid-spring (May 1976) [43] and a single summer period (July 2009) [44,45]. Studies in Greece spanned late spring to fall (May–October), though with gaps in coverage between 2001 and 2004 [15,46]. In Spain, research targeted breeding seasons (2000–2001 [46]) with some reliance on observational data [47]. In Algeria, studies concentrated on late summer to fall (August–October) [13,28], while Cyprus was the only location where the pre-breeding period was explicitly observed (2023–2024; Angelidou (unpublished data) [55]).

The current literature identifies four main methods for dietary assessment in Eleonora’s falcon: direct observations, pellet analysis, nest-item examination, and stomach content analysis. While direct observations are often considered the most accurate for determining diet, they are time-consuming, often impractical, and limited by the falcon’s aerial hunting behavior and the small size of insect prey, which make identification at finer taxonomic levels difficult (Angelidou, unpublished data [55]; Oro and Tella [67]). In contrast, collecting prey remains and analyzing pellets offers a more practical and widely used alternative, although this method is not without its biases. Nest-item examination captures only a subset of the diet (specifically, prey delivered to chicks) and may not reflect the adult’s full dietary range. Stomach content analysis, as noted by Siqueira et al. [68], can yield detailed insights into the quantity and quality of ingested taxa but is invasive and ethically challenging. Indirect methods in general are inherently biased, with their accuracy affected by factors such as prey size and taxon, the predator’s feeding behavior, and methodological inconsistencies in data collection (e.g., frequency of sampling or pellet recovery) [69,70,71]. For instance, Falconiformes often tear prey apart, consuming fewer diagnostic remains, particularly of soft-bodied or small prey, which may lead to underrepresentation in pellet analysis [70]. Our findings align with earlier research, confirming notable differences between methods. Pellets not only captured the broadest variety of insect prey but were also the sole method that detected Mantodea and Dermaptera, and the only one applied across all studied countries. These results underline the importance of prioritizing pellet analysis and direct observations in future studies to ensure more accurate and comprehensive dietary assessments.

Geographically, Greece and especially the islands of the Aegean archipelago (north Sporades, Cyclades-Dodecanese, and Crete) are the most well-studied breeding grounds of Eleonora’s falcon in terms of their insect–prey diet, with results based on all four methods of dietary analyses (direct observations, pellet analysis, nest-item examination, and stomach-content examination). Algeria and especially Kef Amor, Oran, Plane Island, Ronde Island, and Rachgoun Island are the second most well-studied breeding grounds of the species in terms of insect–prey diet, with results based on pellet analysis and nest-item examination. Italy, including Lampedusa, the Pelagian Islands, the Sicilian Channel, Pantelleria, Linosa, Lampione, and the Aeolian Islands, is a less studied area regarding the insect–prey diet of Eleonora’s falcon. Research in this region is based on direct observations, pellet analysis, and nest-item examination. Similarly, Spain, encompassing Son Bosc and the Canary Islands (Alegranza, Montaña Clara, Roque del Oeste, Graciosa, and Roque del Este), is also under-researched in this context. Findings from this area rely primarily on direct observations and pellet analysis. Cyprus has limited data on Eleonora’s falcons’ insect feeding habits, with only one study by Vaughan [51] and subsequent unpublished data [55] contributing to the existing knowledge. No published data on the insect-prey diet of Eleonora’s falcon were found for Morocco, Tunisia, Turkey, Croatia, or Portugal; thus, further studies are necessary.

The studies from Italy, Greece, Spain, and Algeria indicate that the diet of Eleonora’s falcon is diverse and emphasize that this variation is influenced by its breeding status [28]. The present dietary study reveals the dominance of the insect orders Coleoptera, Hymenoptera, and Hemiptera. Scarabaeidae, Buprestidae, Carabidae, Chrysomelidae, Brentidae, Curculionidae, Elateridae, Silphidae, Staphylinidae, and Tenebrionidae are some of the Coleoptera’ families that Eleonora’s falcon consumes. In addition, Hymenoptera were represented essentially by the Formicidae family, such as *Camponotus* spp*., Messor* spp*., Monomorium* spp*., Pheidole* spp., and *Tetramorium* spp., but also species of the order Apidae and Vespidae, with Spina (1992) highlighting that the species hunts ants as a food supplement when larger prey are scarce. Hemiptera was represented primarily by Cicadidae, and to a lesser extent by Coreidae, Cydnidae, Lygaeidae, Miridae, Pentatomidae, and Reduviidae. Odonata, Orthoptera, Lepidoptera, Diptera, Mantodea, and Dermaptera are also prey for Eleonora’s falcon. It is also noteworthy that some of the insect prey we identified are well known for their migratory habits, for example, the moth species of the genus *Acherontia* (i.e. *Acherontia atropos*), a resident species in North Africa and regular summer migrant to Europe, and dragonflies of the genus *Anax* (i.e. *A. parthenope* and *A. ephippiger*) and *Sympetrum* (i.e. *Sympetrum fonscolombii*).

Conducting prey analysis at a regional and international scale is important for detecting dietary patterns and identifying potential mismatches between Eleonora’s falcon’s breeding activities and food supply [15]. However, to gain a more accurate understanding of dietary patterns, further studies are necessary, as the current data may not fully capture the ecological trends. While analyzing diets based on habitat types could provide valuable insights into the relationship between prey availability and habitat-specific foraging, the current literature lacks sufficient data to support a comprehensive analysis in this area. This gap underscores the need for additional studies, particularly given the species’ high mobility. Eleonora’s falcon is known to forage across diverse environments, often traveling hundreds of kilometers from the breeding colony to locate food. Consequently, insect prey identified in diet samples may originate from a range of distant habitats, complicating efforts to infer habitat use without direct observations of foraging behavior.

It is also important to recognize that the methodologies commonly used to study the falcon’s diet, such as analysis of prey remains, pellets, or stomach contents, do not allow for the precise determination of the habitat in which prey was captured. Moreover, trophic diversity may be influenced by land-use changes that alter prey abundance and availability, potentially affecting the species’ population dynamics [72]. Unfortunately, such information remains largely absent from the current literature.

The current systematic review also emphasized that research on the feeding capacity and dietary habits of Eleonora’s falcon is lacking in certain Mediterranean countries (e.g., Croatia, Tunisia, Turkey), or relevant data are not publicly available, or were not captured by our search strategy and could thus not be incorporated. This gap in knowledge is not necessarily due to a lack of ecological importance or interest in the species but is probably attributed to the absence of dedicated experts and/or specialized research teams working in these regions. As a result, the available data may present an incomplete or skewed picture of the falcon’s trophic interactions across its breeding range, potentially limiting the generalizability of findings and the ability to draw comprehensive conclusions. This geographic bias highlights the need for broader research coverage and collaboration in underrepresented areas to ensure a more balanced and holistic understanding of the falcon’s dietary ecology and its role in Mediterranean ecosystems. Addressing this bias could enhance conservation strategies and provide more robust data for future comparative studies.

Moreover, creating a detailed inventory of the falcon’s insect prey throughout the breeding season and assessing dietary variations between colonies at different latitudes across the Mediterranean Sea and the North Atlantic Ocean is essential. Furthermore, additional research is needed to assess the biomass of insects as well as Eleonora’s falcon diet dependency on these insect species. Such studies can help clarify the falcon’s ecological role and its dependence on specific insect species. Moreover, variations in diet across latitudes or over time can serve as indicators of broader ecological changes, including habitat degradation and the effects of climate change on insect populations [13]. Identifying key prey species and their availability, as well as the key priority areas, can inform habitat management strategies, such as preserving or restoring habitats that support critical insect populations, particularly in breeding areas where prey availability directly influences reproductive success [36,37].

Since the 1950s, migrating birds of prey have served as important indicators of the ecological health of both natural and human-impacted landscapes, making them valuable tools for assessing environmental change [73]. Eleonora’s falcon could function as a biological indicator to assess environmental change, as long-term monitoring of the species at several watch-sites has provided valuable data and could significantly enhance our understanding of the impact of large-scale human activities on ecosystems worldwide.

As recommended by Ristow [46], a first step would be to carry out experiments on insect diet preferences as a pellet study in a falcon rehabilitation center. Furthermore, focused efforts to analyze insect–prey at breeding grounds are necessary, as this would be invaluable for identifying dietary patterns and detecting any mismatches between Eleonora’s falcon’s breeding activity and food availability [15]; however, the availability of prey pellets could be a key limitation. For example, in areas like Cyprus, where the availability of prey pellets is limited, research can be hindered.

Alternatively, insect populations, which are key components of the falcon’s diet as highlighted in the current literature, could be investigated through field surveys at known feeding hotspots. A combination of methods is recommended, including direct observations, the use of malaise traps (suitable for monitoring flying insects such as Diptera, Hymenoptera, Hemiptera, and Lepidoptera), line transects, and pellet analysis. In addition, a combined logger and diet method could be applied [74]. More specifically, that could be achieved by monitoring the foraging altitudes of Eleonora’s falcon, by equipping nesting falcons with altitude loggers, and gathering the prey they bring to their nestlings. This will also enable us to determine the flight altitudes of the insects. Combinatorically, the dietary composition of breeding Eleonora’s falcons could be recorded by the use of camera-traps [12]. The population density of Eleonora’s falcons also impacts study feasibility, with higher population densities offering more data collection opportunities. Thus, future studies should always estimate the studied population to account for these challenges.

Conservation of migratory species is challenging because it entails understanding the ecological requirements of individuals living in two geographically separated regions [62]. Migratory raptors are sensitive to climate change [73], so are the Mediterranean ecosystems [75]; therefore, it is essential to prioritize conservation actions of the Mediterranean colonies of the Eleonora’s falcon. Changes in abiotic conditions influence the life histories of organisms across all trophic levels, disrupting biotic interactions, community stability, and ecosystem functioning [76]. Consequently, alterations in the seasonal activity of one species can impact other interacting species, both within and across trophic levels [76]. Raptors, including Eleonora’s falcon, experience the effects of climate change in numerous ways. These include shifts in their distribution ranges, changes in disease and parasite dynamics, alterations in breeding schedules and migration patterns, and fluctuations in population sizes [77]. Additionally, they encompass modifications to community structures and adaptations in their physical traits, physiological processes, and behaviors [77]. Insects, as poikilothermic organisms, are also particularly sensitive to climate change. Their documented responses to climate change include shifts in distribution ranges, local adaptations in thermal tolerance, changes in body size, and altered phenology [76]. Thus, these changes likely impact Eleonora’s falcon, especially since species across different trophic levels can have different response rates to climate change. However, it is important to keep in mind that these drivers of change may not necessarily pose a significant threat to the species, as migratory birds are remarkable examples of adaptability, with many species migrating to temperate regions during the winter months in search of more favorable environments with abundant food resources [78].

## 5 Conclusions

In this review, records of foraging behavior of Eleonora’s falcon (*F. eleonorae*) during the pre-breeding and breeding periods were systematically searched following the PRISMA 2020 guidelines in four online databases. There were 18 studies available on this subject, published between January 1938 and September 2024; however, not all of these studies specifically concentrated on Eleonora’s falcon’s insect-based diet. Nevertheless, 47 insect families and 120 insect species were recorded, and information about the area–location–country and the period of the study were provided. We found that Coleoptera, Hymenoptera, and Hemiptera were the most recorded insect orders and highlighted patterns of similarity in insect family and species composition across Mediterranean regions and countries. Selected records in this review varied in methodology, sample size, and diagnostic methods. Most studies were carried out through observations, pellet and stomach-content analysis. The pellet analysis was the method that revealed the greatest variety of insect prey. Greece was the most extensively studied breeding ground of Eleonora’s falcon in terms of the insect-based diet of the species, encompassing all four dietary analysis methods. This review can serve as a compendium for future dietary ecology studies on Eleonora’s falcon during the pre-breeding and breeding periods of the species. It also highlights the importance of aerial predators as indicators for the monitoring of possible changes in insect diversity across its breeding range.

## Supplementary Material

Supplementary Table
